# The System of Self-Consistent Models: QSAR Analysis of Drug-Induced Liver Toxicity

**DOI:** 10.3390/toxics11050419

**Published:** 2023-04-29

**Authors:** Alla P. Toropova, Andrey A. Toropov, Alessandra Roncaglioni, Emilio Benfenati

**Affiliations:** Istituto di Ricerche Farmacologiche Mario Negri IRCCS, Via Mario Negri 2, 20156 Milano, Italy; andrey.toropov@marionegri.it (A.A.T.); alessandra.roncaglioni@marionegri.it (A.R.)

**Keywords:** drug-induced liver injuries, hepatotoxicity, Monte Carlo method, index of ideality of correlation (IIC), CORAL software

## Abstract

Removing a drug-like substance that can cause drug-induced liver injury from the drug discovery process is a significant task for medicinal chemistry. In silico models can facilitate this process. Semi-correlation is an approach to building in silico models representing the prediction in the active (1)—inactive (0) format. The so-called system of self-consistent models has been suggested as an approach for two tasks: (i) building up a model and (ii) estimating its predictive potential. However, this approach has been tested so far for regression models. Here, the approach is applied to building up and estimating a categorical hepatotoxicity model using the CORAL software. This new process yields good results: sensitivity = 0.77, specificity = 0.75, accuracy = 0.76, and Matthew correlation coefficient = 0.51 (all compounds) and sensitivity = 0.83, specificity = 0.81, accuracy = 0.83 and Matthew correlation coefficient = 0.63 (validation set).

## 1. Introduction

The liver is highly susceptible to drug insults: around 5–10% of adverse drug reactions result in liver injuries [1]. Naturally, this stimulates the search for reliable models to anticipate and avoid this dangerous toxicity [2]. More than 1100 chemical substances applied daily have been identified as potentially causing liver injuries [3,4,5]. The clinical impact may be vary, provoking oxidative stress, an increase in the level of liver enzymes (cytochromes P450), and a dangerous impact on metabolism [5,6,7].

In silico models can help predict adverse effects and plan safer drugs before their complete development. Of course, these models have limits. This is a general issue since experimental studies also have limits of different types, such as the time and costs needed and ethical concerns regarding the use of animals.

“The idea of approximation dominates all exact science” (Bertrand Russell). Quantitative structure–activity relationships (QSARs) are an example of science where approximation is relevant. QSAR should be considered a surrogate of a real experiment with some limits. Even though “all models are wrong” [8], “some of them are useful” [9]. Therefore, the point is to develop “useful models”. This refers to purpose and ambition, and how far we go with a model. For screening purposes, for instance, models for an initial evaluation are acceptable even if they have greater uncertainty. However, models for the final evaluation require much less uncertainty.

We aim to develop some simple, fast models for the first evaluation of large collections of substances. This is suitable for the endpoint we are addressing in the present case: drug-induced liver injuries (DILI). This relates to many toxicological mechanisms involving complexity. At the basis of our model, as with QSAR models in general, there are data collections with experimental values. These data serve to extract the correct information, but as we said, we must verify that the model is “good”, which is achieved with other data not used to build the model. In practice, the original collection of data is split into a training and a validation set, and avoiding the QSAR model becoming too good depends on the precise distribution of substances between these sets. The present paper describes a study on a group of random models to gain a balanced and robust model representative of multiple conditions. This is achieved with our system of self-consistent models applied to DILI. This study is pertinent from the methodological and practical points of view, as the results can be used both to predict DILI and to assess the reliability of the approach employed to build up the group of models.

## 2. Materials and Methods

The experimental data (n = 1274) on DILI in the active (denoted by 1) and inactive formats (denoted by 0) were taken from the literature [1]. The compounds were randomly distributed into active training (≈25%), passive training (≈25%), calibration (≈25%), and validation sets (≈25%). Each set has a defined task. The active training set is used to build the model: molecular features extracted from SMILES of the active training set are used in Monte Carlo optimization with the CORAL software (http://www.insilico.eu/coral) (accessed on 24 April 2023) to provide correlation weights (CW) for these features, which give the largest correlation coefficient between descriptors (the sum of the CW) and the endpoint of the active training set. The passive training set serves to check whether the model for the active training set is satisfactory for substances that were not involved in the active training set. The calibration set should detect the start of overtraining (or overfitting). At the beginning of the optimization, the correlation coefficients between the experimental values of the endpoint and the descriptor rise for all sets, but the correlation coefficient for the calibration set reaches the maximum (this is the start of the overtraining), and further optimization leads to a decrease in the correlation coefficient for the calibration set. The optimization should be stopped when overtraining starts. 

After stopping the Monte Carlo optimization procedure, the validation set is used to assess the predictive potential of the model. The present study applied semi-correlations [10,11,12]. The essence of this approach is the construction of a regression model for a set of compounds characterized by 1 (if the compound is active) or 0 (if the compound is inactive). Ideally, all active and inactive ones are above the regression line.

### 2.1. Optimal SMILES-Based Descriptors 

The optimal descriptor forms the basis of the model suggested here. The descriptor is calculated as follows:

(1) $$DCW\left(T^{},N^{}\right)=\sum_{}^{}CW(S_{k})+\sum_{}^{}CW({SS}_{k})+\sum_{}^{}CW({SSS}_{k})$$

*T* is an integer to separate SMILES attributes into rare and non-rare. The non-rare SMILES are applied to build up the model. The rare SMILES are not used for this. The selection of a value for *T* is empirical, according to the results of preliminary probes of building up the model (usually, it can be 1, 2, or 3). *N* is the number of epochs of the correlation weight (*CW*) optimization. *S_k_* is a SMILES atom, i.e., one symbol of a SMILES line (e.g., ‘=’, ‘O’) or a group of symbols that cannot be examined separately (e.g., ‘Cu’, ‘%11’). *CW*(*S_k_*) are the correlation weights of the SMILES attributes. *SS_k_* and *SSS_k_* are compositions of, respectively, two and three SMILES atoms. *CW*(*SS_k_*) and *CW*(*SSS_k_*) are the correlation weights of the SMILES fragments. The *CW* are obtained through the Monte Carlo method [10,11,12].

The optimal SMILES-based descriptor *DCW*(*T*,*N*) is applied for a model to predict DILI using the equation:

(2) $$y=C_{0}+C_{1}\times DCW(T,N)$$

*y* defines the category of a compound:

(3) $$Category(SMILES)=\left\{\begin{array}{cc} 1(active) & if,y\geq 0.5\\ 0(inactive) & if,y<0.5 \end{array}\right.$$

### 2.2. Monte Carlo Optimization

Equation (1) needs the numerical data on the *CW,* and the Monte Carlo optimization is used to calculate these. Here, two target functions (*TF*_0_ and *TF*_1_) for the Monte Carlo optimization are examined:

(4) $${TF}_{0}=r_{AT}+r_{PT}-\left|r_{AT}-r_{PT}\right|\times 0.1$$

(5) $${TF}_{1}={TF}_{0}+{IIC}_{C}\times 0.5$$

$r_{AT}$ and $r_{PT}$ are correlation coefficients between the observed and predicted endpoints for the active and passive training sets. *IIC_C_* is the index of ideality of correlation [13]. Recent computational experiments have shown [13] that considering the *IICc*-value in the Monte Carlo optimization may be useful. *IIC_C_* is calculated with data on the calibration set as follows:

(6) $${IIC}_{C}=r_{C}\frac{min({}^{-}{MAE}_{C},{}^{+}{MAE}_{C})}{max({}^{-}{MAE}_{C},{}^{+}{MAE}_{C})}$$

(7) $$\min\left(x,y\right)=\left\{\begin{array}{c} x,if\;x<y\\ y,otherwise \end{array}\right.$$

(8) $$\max\left(x,y\right)=\left\{\begin{array}{c} x,if\;x>y\\ y,otherwise \end{array}\right.$$

(9) MAEC−=1N−∑∆k,N is the number of ∆k−<0

(10) MAEC+=1N+∑∆k,N is the number of ∆k+≥0

(11) $$\Delta_{k}={observed}_{k}-{calculated}_{k}$$

The corresponding formulae accompany all values; *r_c_* is the correlation coefficient between the observed and calculated values of the endpoint in the calibration set and ‘*c*’ indicates that it belongs to the calibration set. Observed and calculated are the corresponding values of *y*. 

### 2.3. The System of Self-Consistent Models

The system of self-consistent models [14] for five random splits into the training (visible) and validation (invisible) sets confirms the high quality (predictive potential) of the models. The training set here is divided into active, passive training, and calibration sets. Thus, the difference between models reflects the difference in training sets. However, the key attribute of the system of self-consistent models is the unified method for validating these models; each *i*th model has an *i*th validation set. The validation sets are far from identical (Table S1, Supplementary Materials). This supports the statistical fact that we explore multiple conditions, and the results are representative of a set of cases, each obtained by chance, and their overall results should be evaluated jointly.

The measure of self-consistency is based on the average and dispersion of the Matthews correlation coefficient (*MCC*) in different validation sets. The corresponding computational experiments are represented by the following matrix:

(12) $$\left[\begin{array}{ccc} \left(M_{1}(V_{1}):V_{1}\to {MCCv}_{11}^{}\right) & \cdots & {(M}_{5}(V_{5}):{V'}_{1}\to {MCCv}_{51}^{})\\ \vdots & & \vdots \\ {(M}_{1}(V_{1}):{V'}_{5}\to {MCCv}_{15}^{}) & \cdots & \left(M_{5}(V_{5}):V_{5}\to {MCCv}_{55}^{}\right) \end{array}\right]$$

$M_{i}$ is an *i*th model, ${V'}_{j}$ is the list of compounds employed as the validation set in the case of the *j*th split, and ${{MCCv}_{ij}^{}}_{}$ is the Matthews correlation coefficient for the *j*th validation set if applied to the *i*th model. Figure S1, in the Supplementary Materials, shows the general scheme of validation of model 1 with validation set 3. 

## 3. Results

**Figure 1:**
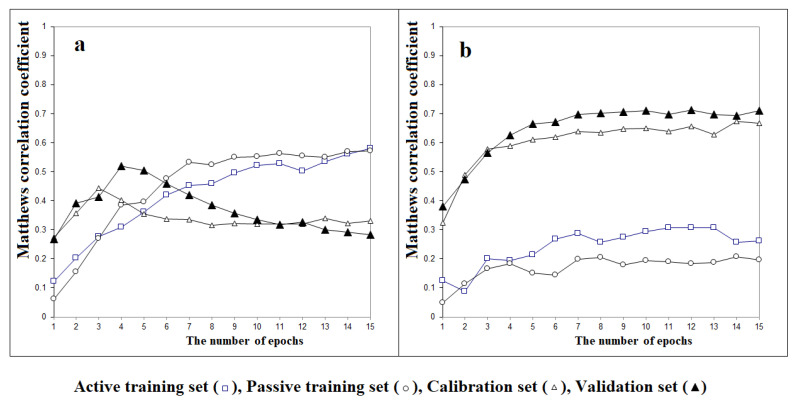
The evolution histories of the Matthews correlation coefficients during the Monte Carlo optimization for the active training set, the passive training set, the calibration set, and the validation set: (**a**) the optimization without *IIC* and (**b**) the optimization with *IIC*.

We applied different methods using the CORAL software. Some models were developed with the classical approach, as in Equation (4), while others used a more recent approach, using Equation (5). Figure 1 shows the pattern of the Monte Carlo optimization of the correlation coefficient between the experimental and calculated *y*-values for the active training, passive training, calibration, and validation sets in the case of the Monte Carlo optimization without *IIC* (Figure 1a) or optimized with *IIC* (Figure 1b).

The determination coefficients of the training set increase slowly and continuously. Nevertheless, the patterns for the values of the other two sets, calibration and validation, are different, in particular for the validation. In Figure 1a, they peak earlier. As a consequence, depending on the epoch number, the results vary. The values for the two training sets (passive and active) are not a good indicator of the results when new substances are predicted. On increasing the number of epochs, overfitting starts (Figure 1a). It is also clear that in our conditions, the training set values often differ from those of the other sets, and depending on the epoch, the highest or lowest values of the training sets appear.

Figure 1b shows the Matthews correlation coefficients for the active training, passive training, calibration, and validation sets in the case of the optimization with *IIC* (Equation (5)). In this case, the training set provides general features that are useful for the model, and the calibration set optimizes these features. The overall process is successful since the results for the validation set in Figure 1b are better than those in Figure 1a.

Table 1 illustrates the acceptable statistical quality of the approach, with the reproducibility of the predictive potential for all variations, expressed as

(13) $$M_{i}(V_{i}):{V'}_{j}\to {MCCv}_{ij}^{},\;i\neq j$$

since the average ${\overset{-}{MCC}v}_{ij}^{}$ = 0.7634 ± 0.0528. 

The comparison of the models described here with the statistical quality of DILI models from the literature (Table 2) confirms the practical and heuristic potential of the developed approach. 

## 4. Discussion

The approach considered here is based on the use of semi-correlations. The latter are specific kinds of correlations where one variable takes only two values (for example, 0 and 1), expressing the presence (1) or the absence (0) of some modeled activity. The concept of categorical (binary classification) simulation has been successfully tested for several types of biological activity [15,16,17,18,19,20,21]. The convenience of the practical application of the concept of semi-correlation lies in the possibility of using SMILES to represent the molecular structure without requiring additional descriptors. The hidden analogy of semi-correlations with the usual linear regression helps the perception and interpretation of the resulting models. The necessary CORAL software is available on the internet (http://www.insilico.eu/coral) (accessed on 24 April 2023). The models considered here are comparable in their predictive potential with models obtained through more complex calculations using random forest procedures, support vector machines, gradient methods, and others [2,3,4,5,6].

Of particular note are actually new principles for testing the predictive potential of models that can be used for any similar models aimed at developing categorical binary models of biological activity. Criticism of cross-validation has a long history. The essence of claims of Q^2^ is its weak relationship with the predictive ability of models [22]. The ‘naïve’ Q^2^ [22] is present as a characteristic of the model; however, this criterion has lost confidence. The first attempt to achieve a plausible test of the predictive potential of the model was to use an external set of test substances unknown at the time of model development called the validation set [23]. However, if we assume that the model can be built on an arbitrary distribution of data in the training and testing sets, then any model being constructed based on a random split should be considered as some random event described by statistical criteria (most significant ones, are related to the validation set) [24].

In view of what has been said, the system of self-consistent models looks very attractive from the point of view of justifying the use of an appropriate approach to constructing the models. A test of this scheme for the case of ordinary regression models showed the convenience of using the approach for linear regression models [14,25,26,27,28,29]. Since the system of self-consistent models implies the consideration of groups of models with the allocation of average values of statistical parameters, this approach provides more objective information about the statistical significance of the approach used.

The index of ideality of correlations is an important component of the considered scheme for constructing self-consistent models. This criterion for the predictive potential of linear regression models has found a number of applications for the development of various endpoints related to organic substances [30,31,32,33,34], polymers [26], and nanomaterials [35,36,37,38,39,40]. The universality and attractiveness of *IIC* applications are probably due to the fact that this index contains information on correlation and average absolute error. It should be noted that the self-consistency methodology of models and the use of *IIC* are two innovations in modeling that harmoniously complement each other.

Thus, the predictive potential of corresponding models is similar for all random splits. However, the model applying the *IIC*, i.e., the Monte Carlo optimization using the target function calculated with Equation (5), is better. Table 2 compares models for the DILI reported in the literature.

The advantage of our model is that it is simple: it does not require the calculation of molecular descriptors since only the SMILES are used. The results are optimized towards the prediction of new substances, remaining far from overfitting. Even if this may reduce the results of the training set, it is a good approach for predicting other substances. The detection of outliers for models based on semi-correlations is carried out using the values of statistical defects described in the literature [10].

## 5. Conclusions

We have introduced some new models for DILI using the CORAL software. The statistical quality of models developed here confirms (i) the suitability of the index of ideality of correlation as the criterion for the predictive potential and (ii) the expediency of the system of self-consistent models as the rational method for the validation of QSAR models for DILI. 

## Figures and Tables

**Table 1:** The statistical characteristics of the DILI model observed for split 1.

Set	Observed Classification Quality					Statistical Characteristics			
	TP	TN	FP	FN	N	Sensitivity	Specificity	Accuracy	MCC
Active training	119	99	49	47	314	0.7169	0.6689	0.6983	0.3861
Passive training	123	101	32	63	319	0.6613	0.7594	0.7022	0.4150
Calibration	160	100	28	31	319	0.8377	0.7813	0.8150	0.6167
Validation	181	86	20	35	322	0.8380	0.8113	0.8292	0.6300
Total	583	386	129	176	1274	0.7681	0.7495	0.7606	0.5116

**Table 2:** The statistical quality of the DILI models reported in the literature.

N	Sensitivity	Specificity	Accuracy	Sensitivity (Validation Set)	Specificity (Validation Set)	References
-	0.73	0.73	-	-	-	[2]
6853	0.91	0.53	0.79	-	-	[3]
1550	0.76	0.71–0.92	-	-	-	[4]
1148	-	-	-	0.68–0.76	0.83–0.99	[4]
1254	0.82	0.75	0.78	-	-	[5]
83	-	-	-	0.818	0.748	[5]
1036	0.82–0.90	0.55–0.64	0.71–0.75	-	-	[6]
1274	0.77	0.75	0.76	-	-	This work (split 1)
322	-	-	-	0.838	0.8113	This work (split 1)

## Data Availability

Not applicable.

## Supplementary Materials

The following supporting information can be downloaded at: https://www.mdpi.com/article/10.3390/toxics11050419/s1, Table S1 contains MCC values for validation set after removing compounds present in the training set; and Table S2 contains technical details on split 1. Figure S1 demonstrated the general scheme of checking up of model-1 with validation set-3.
